# Applying a semi-quantitative risk assessment on petroleum production unit

**DOI:** 10.1038/s41598-024-57600-2

**Published:** 2024-03-31

**Authors:** Fatma M. Eltahan, Monica Toderas, Moustapha S. Mansour, El Sayed Z. El-Ashtoukhy, Mohamed A. Abdou, F. Shokry

**Affiliations:** 1 Chemical Engineering Department, Faculty of Engineering, Alexandria University, Alexandria, Egypt; 2 Department of Physics, Faculty of Sciences, University of Oradea, University St. No. 1, 410087 Oradea, Romania; 3 Informatics Research Institute (IRI), City for Scientific Research and Technology Application (SRTA-City), Alexandria, Egypt; 4 Chemical Engineering Department, Faculty of Engineering, Portsaid University, Port Said, Egypt

**Keywords:** LOPA, HAZOP, Risk assesment, QRA, Petroleum industry, Chemical engineering, Risk factors

## Abstract

Applying safety means in the industry, especially in the petroleum industry is very important to maintain the industrial facility. A semi-quantitative risk assessment as Layers of Protection Analysis (LOPA) is used widely to quantify data after qualitative risk analysis as HAZOP using a simpler way than quantitative risk analysis ‘QRA’ as fault tree analysis ‘FTA’. This determines if a new safety integrity function ‘SIF’ is needed. This paper introduces a novel fuzzy logic system to solve the failure of crude oil shipping pumps. Several models are studied to select the most appropriate fuzzy membership functions. Results are compared with results from the LOPA model, which shows the advantages of using the proposed model to reduce the RRF for the potential hazard and achieve a simple and reliable control method.

## Introduction

Energy consumption is fueled by the expanding global population and the desire to improve quality of life, which results in ongoing energy depletion^1–4^. In countries where there is a great need for fuel, whether for industrial use, energy generation, or even transportation mobilization, crude oil plays a very significant role. Therefore, crude oil is a vital resource principally in industrial settings where it is used in the energy contribution^5,6^. As a result, transportation operations of crude oil from wells to reach the refining stations is a very important stage and while we are dealing with petroleum products that are exposed to the risk of combustion and explosion at any time due to any external cause, strict safety measures should be applied to keep even the individuals or the facility safe and ensuring the success and continuity of facility’s production. Risk is formally defined as the effect of uncertainty on objectives (ISO 31000:2009)^7^. Many risks can be found in industrial facilities due to human error or equipment failure which may cause human injuries, fatalities, environmental damage, or even economic loss. Application of means of safety prevent or mitigate the hazards that are the reason of impact event that cause the harmful consequences.

**Figure 1:**
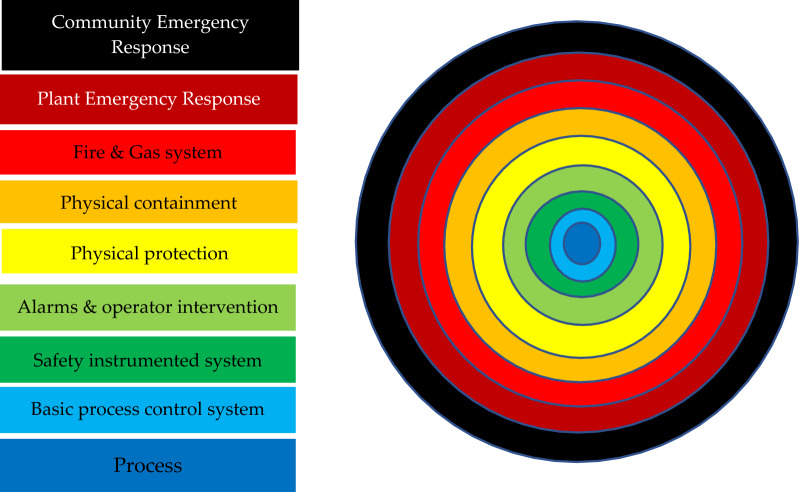
Typical IPLs against potential incidents.

Several risk assessment techniques can be used to identify the potential hazard and its impacts at various stages during the process. HAZOP is one of the famous qualitative analyzing approaches, used from the 1970's, while LOPA receives output from a qualitative hazard analysis such as HAZOP studies and it leads to quantification^8^. The Hazard and operability (HAZOP) technique is one of the Process Hazard Analysis (PHA) techniques used widely for estimating both the hazards of a system, and its operating barriers, by examining the impacts of any deviations from design conditions^9^. While these techniques alone are unable to assess the contributions of particular system components to the overall risk of a chemical facility, further risk assessment methods are studied. LOPA is one of these methods which gives a simple and good performance to analyze risks and prevent or reduce the consequences of undesired cases^10,11^. Layers of protection analysis LOPA is a semi-quantitative method for analyzing and rating risk, as mentioned in the IEC61511 standard^12^, the main aim of applying LOPA is to estimate whether there are enough independent protection layers (IPLs) to mitigate risk to an acceptable level for a selected incident scenario. Typical IPLs are shown in Fig. 1.

Fuzzy logic is a knowledge-based system for quantifying, estimating, and assessing imprecision^13^. The models built using the fuzzy logic approach are reliable, efficient, and cost-effective. To meet the requirements for safety, they could also be modified^14^.

Ouazraoui et al.^15^ represented the data from reliability databases and experts opinions in LOPA using fuzzy quantities. Since no uncertainty information is retained, even though the outcome is a parameter value, this is regarded as a disadvantage of the approach. Khalil et al.^14^ performed a risk assessment research using cascaded Fuzzy-LOPA model, to avoid or lessen undesired events in a natural gas plant, which can handle various frequencies and is employed for specific dangerous situations. The model built calculate the SIL rating through two fuzzy control stages while The proposed system is easier and uses only one fuzzy control stage that calculates TEF, SIL rating is then calculated using a similar method to that in traditional LOPA. Darwish et al.^16^ applied the HAZOP approach to a particular DRI market niche. By keeping an eye on the LOPA study, they sought to gauge the outcomes and assess the system's IPLs for sufficiency by identifying SIL. Also a fuzzy logic is employed for risk assessment to calculate the SIL ratio and compared the estimated frequency to that obtained by conventional LOPA. They guess the use of Gaussian MF in their model while in our study we use three different MFs to decide which one is better. The pre-methods were used in several industries, according to the literature researchs, but not in crude oil shipping facilities. Applying the procedures will therefore greatly aid in providing high levels of protection. This work is concerned with the catastrophic failure that could occur due to reverse flow from the new crude oil pumps’ discharge into the existing crude oil pumps’ discharge and have severe consequences in the shipping system. It presents a fuzzy-LOPA model for SIL assessment that uses the data obtained from the HAZOP along with that from the look-up tables. The proposed system is easier and uses only one fuzzy control stage that calculates TEF. The SIL rating is then calculated using a similar method to that in the LOPA^16^. Since it is difficult to guess the suitable MF to be used, this fuzzy model is based on using the most common MFs to select the better ones with two sets of assumed rules. Finally results from the LOPA model and fuzzy one are compared.

## Hazard and operability study (HAZOP)

HAZOP, which examines the effects of any deviations from design and operating circumstances, is one of the most organized methodologies for analyzing risks and operability issues. It was created in the 1970s by Imperial Chemical Industries (ICI), but it wasn’t until a chemical plant explosion in the UK that was creating nylon intermediate that resulted in 28 deaths and numerous injuries that it really became a standard^17^.

It is a qualitative risk assessment method that can be used for hazard assessment at all stages of the process life cycle, from process development to plant shutdown, as well as for any adjustments that are being considered during normal operation^16^, further, it has the benefit of applying to all types of processes, whether continuous, batch, or semi-batch. Maximum details, such as P&IDs drawings, flowcharts, process descriptions, and for batch/semi-batch operations, an operating manual, must be provided to construct a high level of detailed HAZOP research*.*

**Figure 2:**
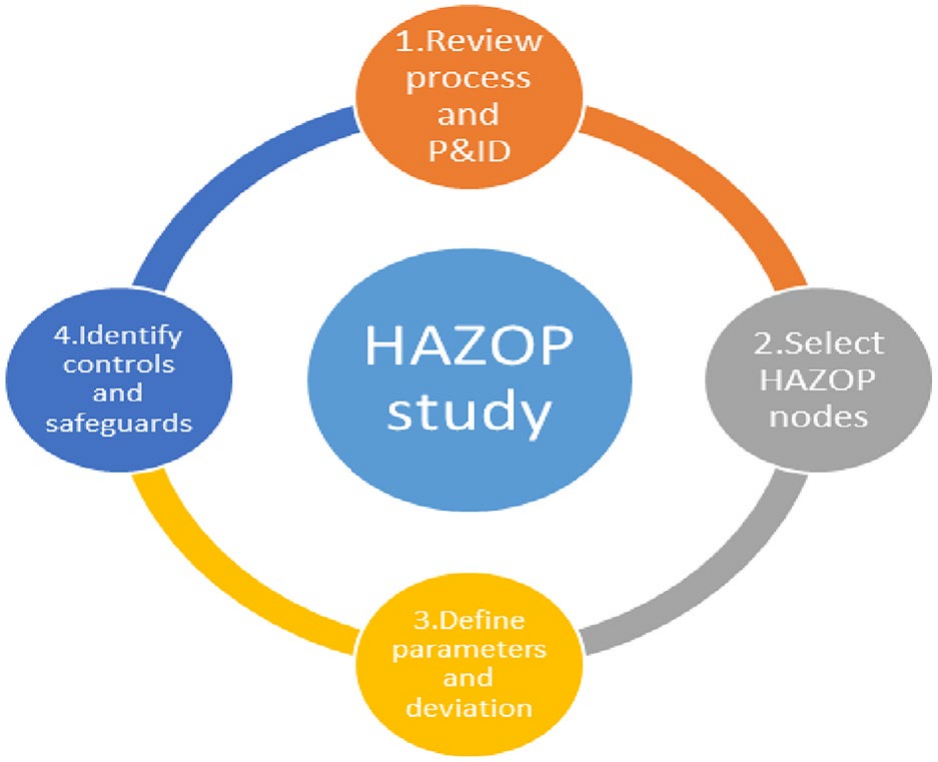
HAZOP study structure.

The HAZOP technique carefully examines plant processes and operations to see whether there might be any deviations from the intended conditions that might materialize during operation due to a particular deviation^16^. Figure 2 represent the HAZOP study structure.

Despite HAZOP technique used widely, it has some limitiation procedure is meticulous and time consuming and is unwarranted for systems where the risk is minor. Instead of using algorithms, it depends on heuristics, and the formal framework provides practitioners the impression that a thorough study is being conducted. There are no assurances that the most significant deviations, triggers, or scenarios have been found, though. Because of this, HAZOP investigations are prone to being insufficient, and this risk grows as the system becomes more complicated and there are more factors to take into account^17^.

## Layers of protection analysis (LOPA)

Despite HAZOP technique used widely, it has some limitiation procedure is meticulous and time consuming and is unwarranted for systems where the risk is minor. Instead of using algorithms, it depends on heuristics, and the formal framework provides practitioners the impression that a thorough study is being conducted. There are no assurances that the most significant deviations, triggers, or scenarios have been found, though. Because of this, HAZOP investigations are prone to being insufficient, and this risk grows as the system becomes more complicated and there are more factors to take into account^16^.

A simple semi-quantitative^18^ hazard analysis method called LOPA can be used following a HAZOP investigation. The parameters required for the computation of the necessary risk reduction level are rated using numerical categories. Safeguards that meet the IPLs (Independent Protection Layers) standards established by the CCPS (Centre of Chemical Process Safety) can be found using LOPA^19^. LOPA supports compliance with process safety regulations-including OSHA PSM1910.119, Seveso II regulations, ANSI / ISAS84.01, IEC61,508, and IEC61511^16^. IPLs can be passive, like a dike, fireproofing, or blast wall, or active, like a relief valve, rupture disc, or BPCS (Basic Process Control System). These crucial requirements for an IPL must exist^20^.

LOPA is applied to a single cause-consequence pair at a time. An acceptable or maximum tolerable risk is compared to the resulting risk. Additional IPLs will be added to the process if the estimated risk of the chosen scenario is considered to be very high which calls for higher installation and maintenance costs.

**Figure 3:**
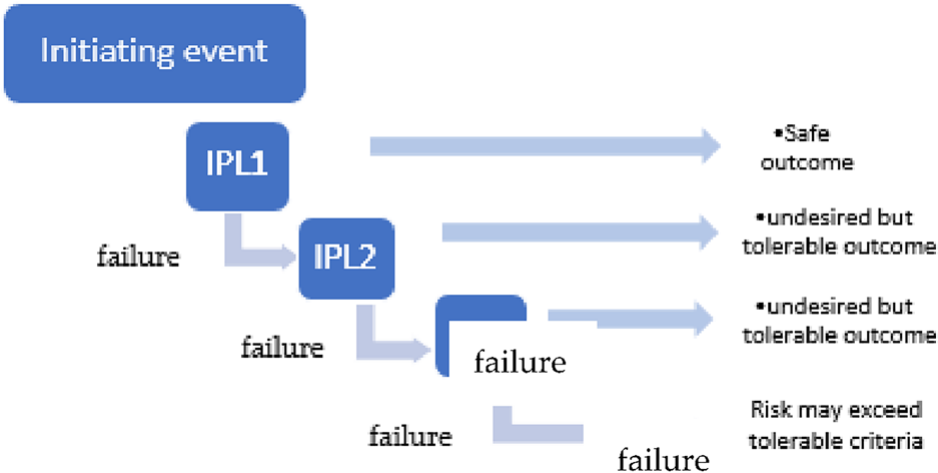
Event tree showing the hazard scenario analyzed in LOPA.

For the selected case identified during a qualitative hazard review such as a HAZOP study risk assessment using the LOPA technique, can be explained as follows:

- Select a failure scenario. LOPA is applied to one scenario at a time. This scenario describes a single-cause consequence pair.
- Identify the initiating event of the scenario and estimate the initiating event frequency. Typical databases are that of the Center for Chemical Process Safety (CCPS)^21^ and the Offshore Reliability Data Handbook (OREDA)^22^.
- List the Independent Protection Layers (IPLs) and calculate each IPL's Probability of Failure upon Demand (PFD). To obtain a tolerable risk for the accident scenario, some accident situations only need one IPL, while others need numerous IPLs or IPLs with a very low PFD as shown in Fig. 3. The essence of LOPA is identifying the safeguards that satisfy the requirements of IPLs for a given scenario. By adding the frequency of the beginning event, the IPL values, and the consequence value, you may estimate the risk of the scenario.

To decide how to respond to the situation, a scenario’s risk must be matched against acceptable risk standards.

An IPL must have the following important characteristics^20,23^:

- *Specificity* Ability to lessen or stop the effects of a potentially harmful occurrence.
- *Independence* Separate from other layers of protection associated with a particular hazard event.
- *Dependability* Must offer protection that lessens the potential risk by a particular percentage.
- *Auditability* It is designed to help ensure that the protective functions are consistently effective.

LOPA technique helps to set the required SIS (Safety Instrumented Systems), and SIL rating.

The SIL rating is shown in Table 1 rated from 1 to 4 indicates the safety protection level wanted in such a process. SIL1 meets the lowest rate of safety protection while SIL4 meets the highest one, which is used only for the nuclear industry^24^.

**Table 1:** SIL rating meeting RRF value.

Safety integrity level (SIL)	Probability of failure on demand (PFD)	Risk reduction factor (RRF) = (PFD-1)
NR	0.00001–0.000001	100,000–1000,000
4	0.0001–0.00001	10,000–100,000
3	0.001–0.0001	1,000–10,000
2	0.01–0.001	100–1,000
1	0.1–00.01	10–100
NSSR	1–0.1	1–10

## Fuzzy logic technique

The Zadeh principle of complexity states that the more complex a system is the less exact information is available^25^. Even though this information is rapidly evolving, there is still much information that is absent and unclear, hidden in variables, models, and subjectivity, particularly when it comes to highly infrequent events like catastrophic disaster dangers. There are numerous methods for analyzing uncertainty, including the possibility approach, sensitivity analysis, classical statistics, and probabilistic methods^26^. There is a widespread belief that using fuzzy logic is considered one of the powerful ways to handle all kinds of information including ignorance and vagueness^27^

The basic concept of fuzzy logic is of a linguistic variable nature as its values depend on words rather than numbers. Words are less precise than numbers; however, they are closer to human intuition^28,29^. Fuzzy Logic techniques are used widely in the field of process safety analysis fault tree analysis, fuzzy risk matrix, bow-tie analysis, etc.^30–45^ also a lot of researches are performed to apply fuzzy LOPA as a new approach to calculate the SIL rating meeting the risk^1,14,16,27^.

**Figure 4:**
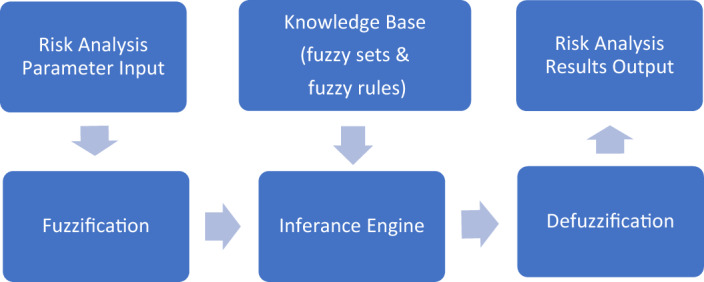
Fuzzy models’ architecture.

The Mamdani procedure^46^ is used to determine the outcome of the membership functions on the relevant variables and derive a final crisp parameter value. It is based on a simple structure of maximum and minimum operations based on fuzzy logic rules cast in IF… Then… statements. The Matlab Fuzzy Logic toolbox can be used to perform fuzzy modeling in three steps, and Fig. 4 interprets the conceptual organization of the modeling system.

### Steps of fuzzy modeling

- Suitable input, output, and discourse universe variables for each variable are fuzzified, and then, fuzzy membership functions count the number of linguistic phrases for each input and output variable.
- The fuzzy inference system employs the Mamdani technique. Based on the information and knowledge that is already available (such as common sense, the knowledge of experts, and physical laws), create a list of fuzzy if–then rules.
- The resulting fuzzy membership function is defuzzified. Convert the output of each fuzzy if–then rule into a crisp value that can be understood.

## Case study

### Node description

The crude oil shipping facility aims to collect crude oil from oil fields by implementing new storing and pumping facilities within plants and a new transporting pipeline.

Crude Oil (C.O) from the 10-TK-011 storage tank is transported through a set of centrifugal pumps with suction and discharge piping system 10-P-001 A/B/C (C.O shipping pumps) have a rated capacity 280 m^3^/hr and diff pressure 45.5 bar.

**Figure 5:**
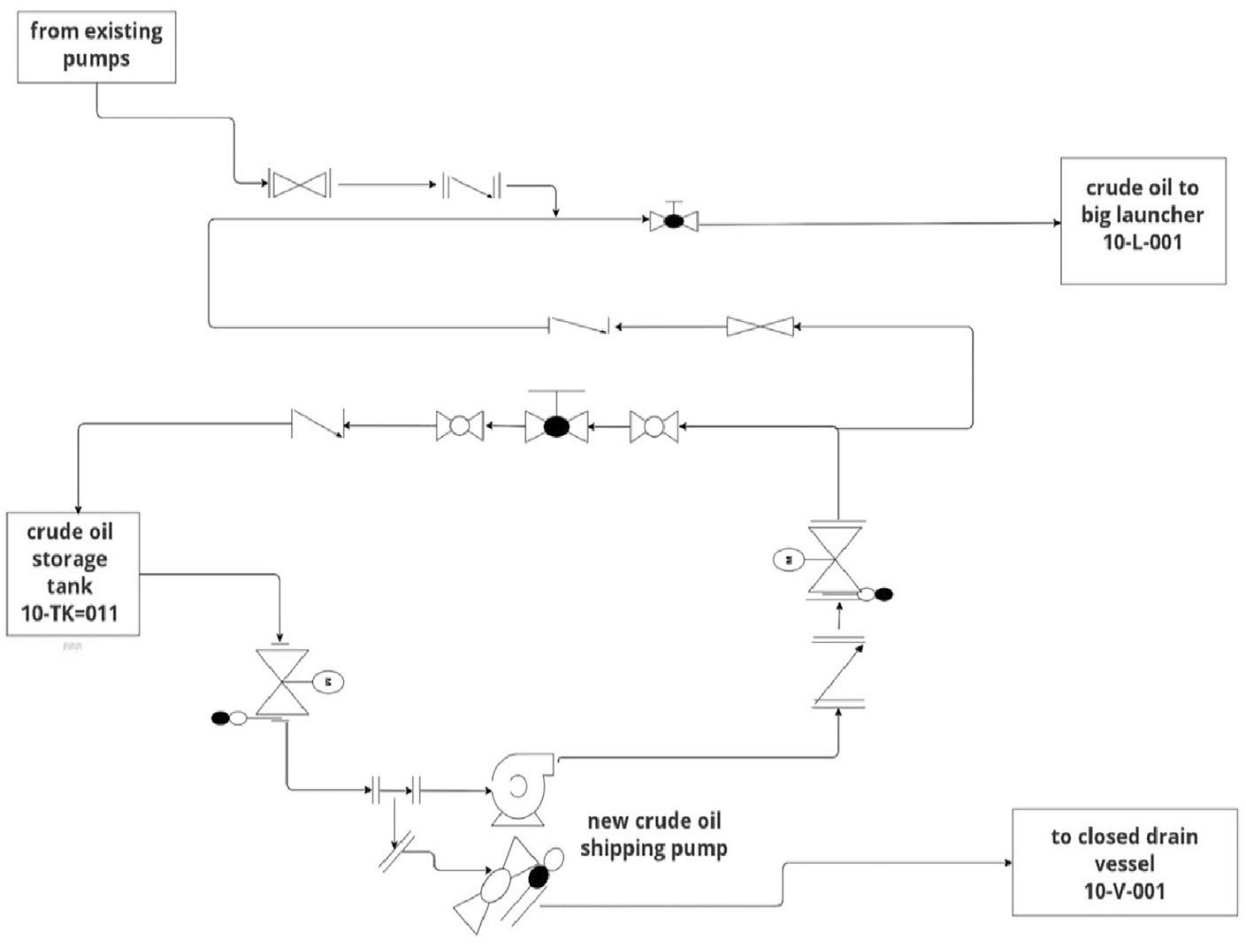
Process flow diagram of new crude oil shipping pumps.

The new shipping pumps output stream is combined with C.O from existing pumps and reaches pig launcher 10-L-001 while a part of the feed from the storage tank go to the closed drain vessel 10-V-001. Figure 5 represents the process flow diagram of the new crude oil shipping pumps.

### Research methodology

**Figure 6:**
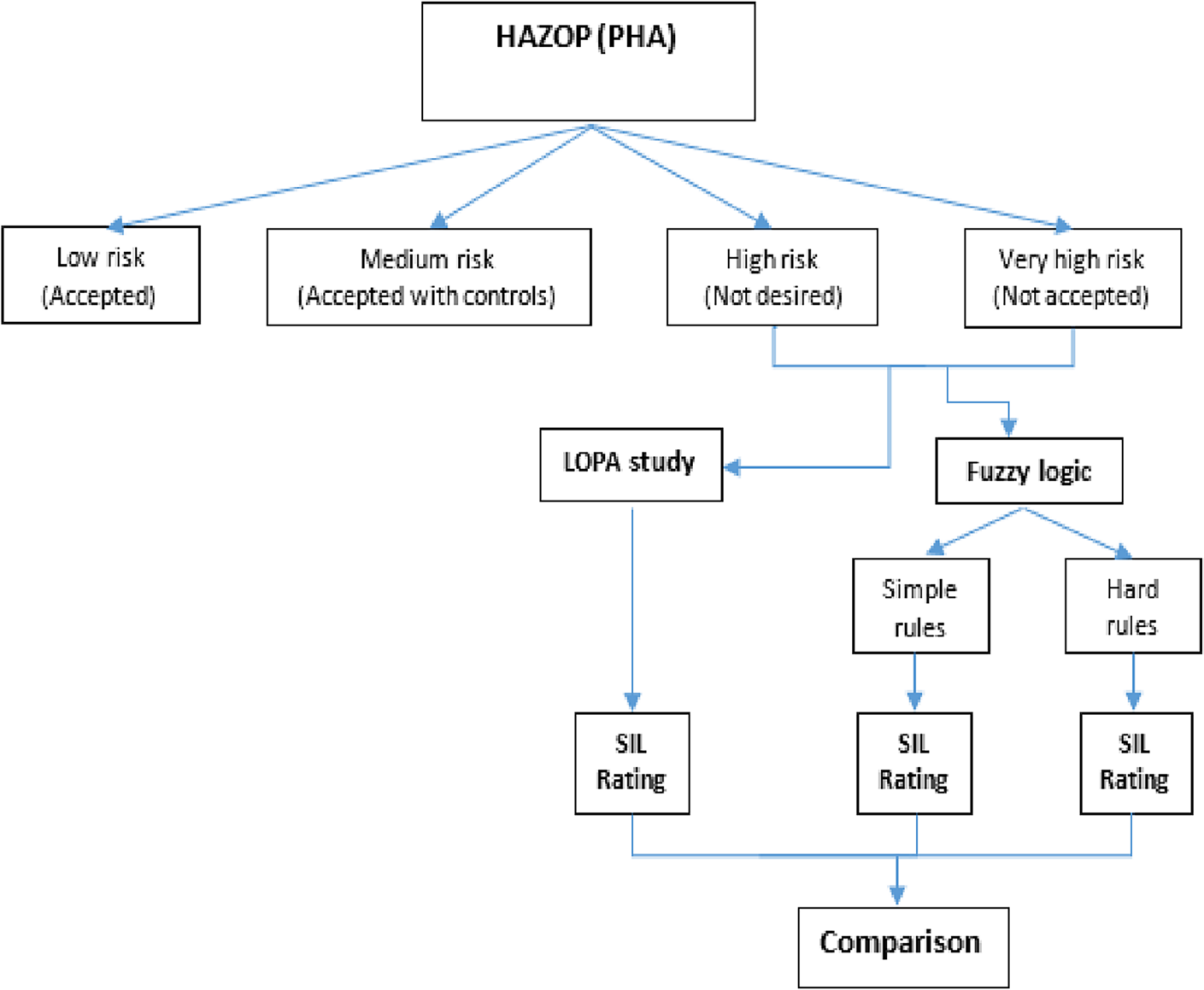
Semi-quantitative risk assessment methodology flow diagram.

In accordance with the CCPS Layer of Protection Analysis recommendations, the research methodology included defining a specific chemical process unit and conducting a PHA to identify potentially dangerous situations, as well as conducting layers of protection analysis on the most dangerous situations shown at Table 2 according to its severity degree shown at Table 3 to determine the SIL requirements for any additional safeguards that may be required. The data collected from the PHA and look-up tables are used with the fuzzy logic model to establish the SIL required for any additional precautions. The results of the LOPA technique and those obtained from the fuzzy model are then compared. Figure 6 depicts our research methods.

**Table 2:** HAZOP table for new crude oil shipping pumps.

Deviation	Causes	Consequences	Safeguards
1. High pressure	1.1. High pressure/low pressure interface (HP/LP) between the new crude oil shipping pumps’ discharge side and the existing crude oil pumps discharge side	1.1.1. Potential for damage within the vulnerable (low pressure) discharge side of the existing crude oil pumps due to overpressure	1.1.1.1. high pressure alarms
2. Reverse/misdirected flow	2.1. Reverse flow from the new crude Oil pumps’ discharge into the existing crude oil pumps’ discharge	2.1.1. Potential for damage within the vulnerable (low pressure) discharge side of the existing crude oil pumps due to overpressure	1.1.1.1. Check valve on existing crude oil pumps’ discharge side 1.1.1.2. high pressure alarms

**Table 3:** Severity degree and TEF.

Severity	Economical lose $	No of injuries	TEF/year
Notable	< 2,000,000 $	First aid case	10^−2^
Minor	2,000,000–20,000,000 $	Medical treatment case	10^−3^
Serious	20,000,000–400,000,000 $	1–2 Lost time injury case	10^−4^
Major	400,000,000–700,000,000 $	3–4 lost time injury cases	10^−5^
Catastrophic	700,000,000–1,000,000,000 $	5–10 lost time injury cases	10^−6^
Disastrous	> 1,000,000,000 $	> 10 lost time injury cases	10^−7^

After determining the SIL rating for the most dangerous scenarios using LOPA study, an additional investigation applying fuzzy logic is carried out to quantify the severity of scenarios and calculate the SIL rating to be compared with the one acquired by LOPA study. In order to use the fuzzy logic technique, the impact events' severity has been modelled using the MATLAB toolbox.

**Figure 7:**
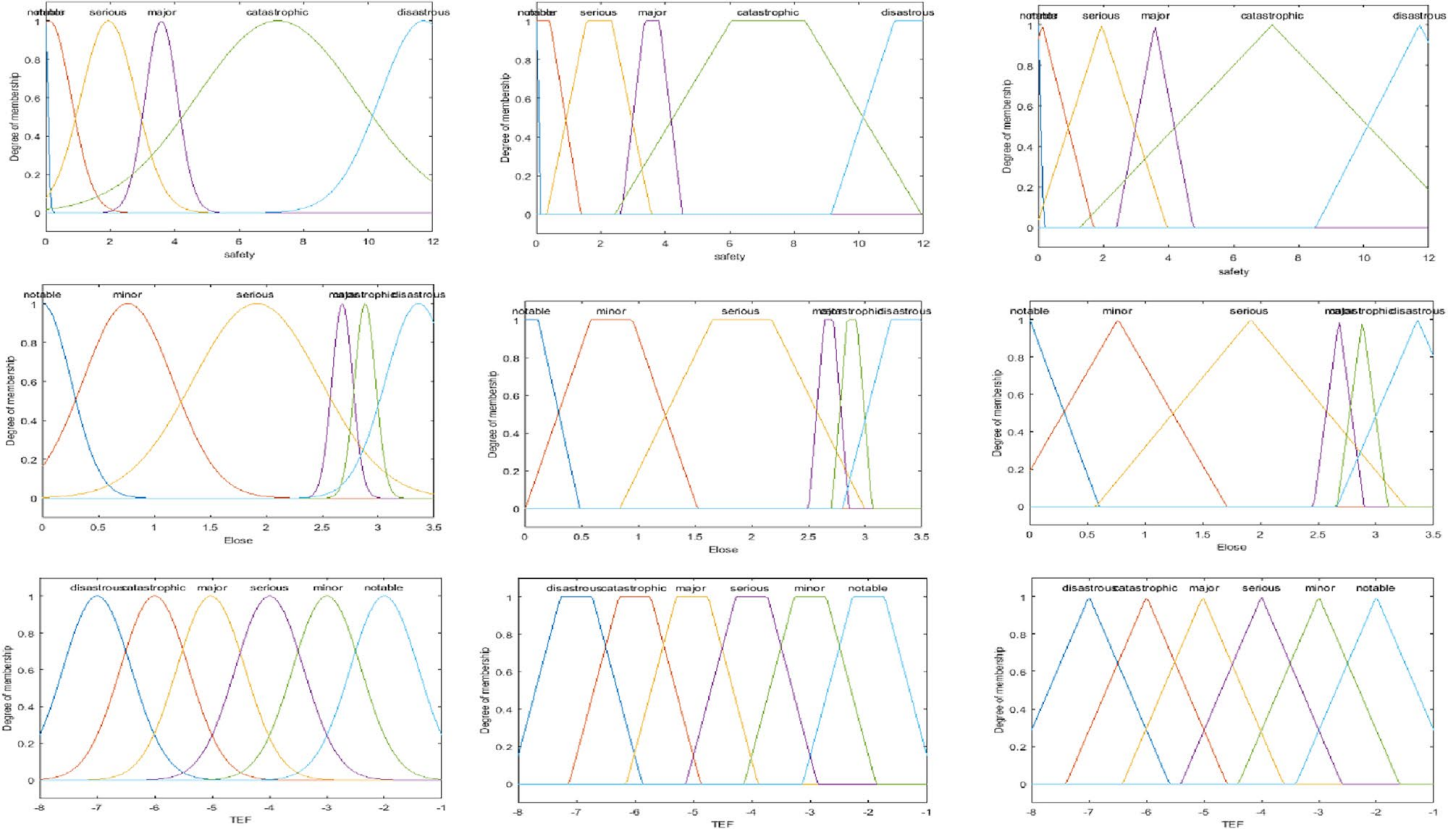
Fuzzy logic variables using different membership functions.

**Figure 8:**
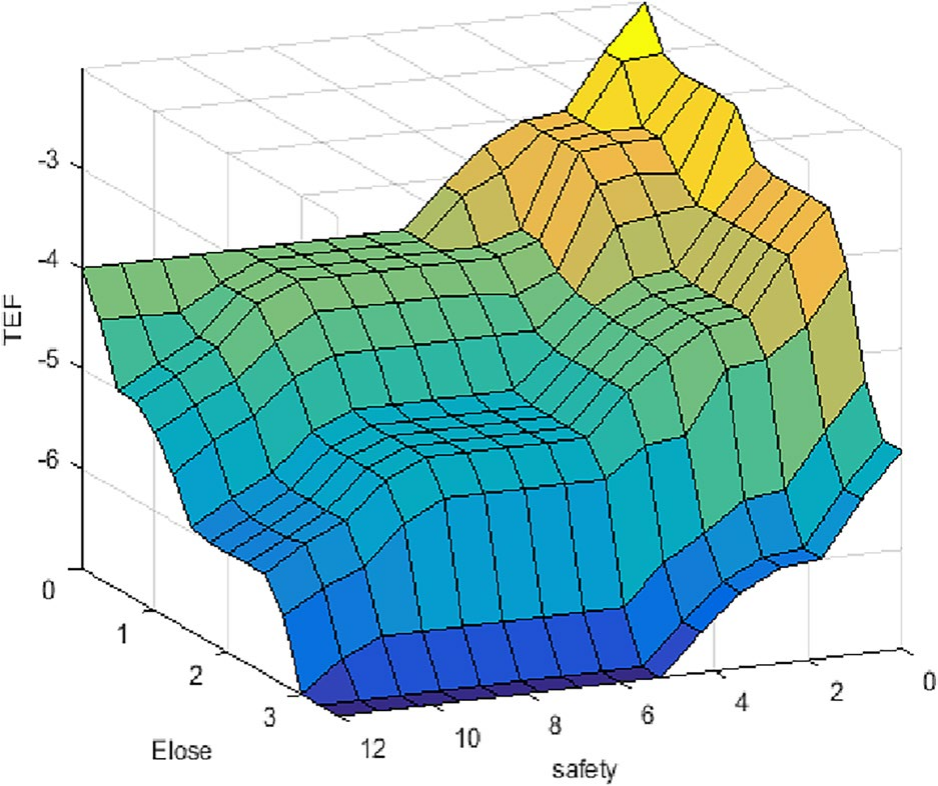
3D surface of hard set of rules.

**Figure 9:**
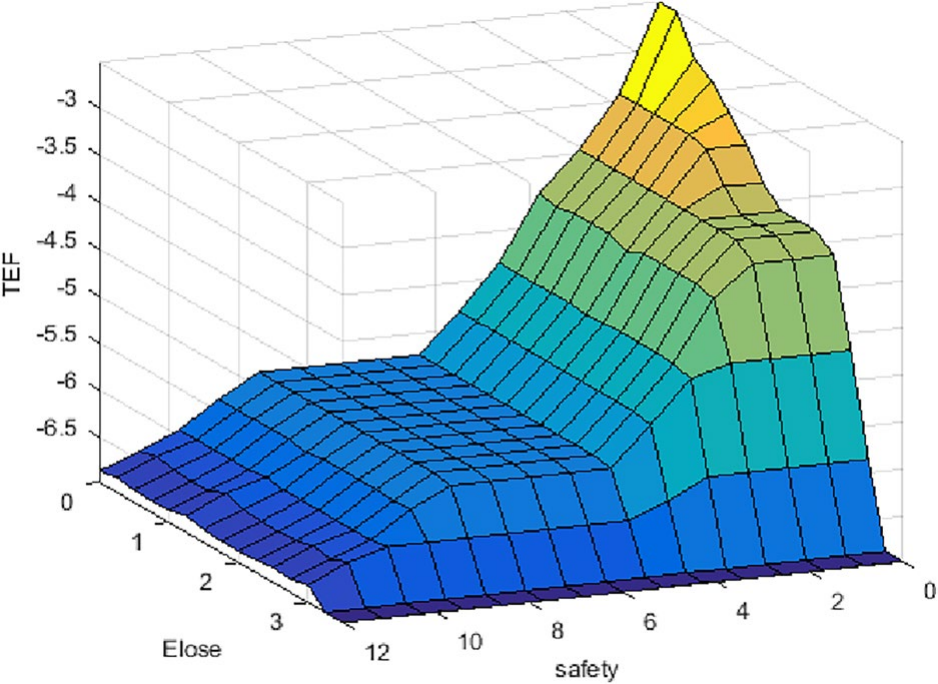
3D surface of hard set of rules.

Our prior experience with fuzzy logic and our analysis of this data result in the described system. The proposed system has one output and two inputs. It is necessary to assign membership functions (MF). To achieve the best results in a fuzzy model, the fuzzy model is built using the most popular MFs, which are Gaussian, trapezoidal, and triangular, with two different sets of rules (simple and hard), as shown in Table 4A, B. Fuzzy logic variables using different membership functions are shown at Fig. 7. Figures 8 and 9 depict the 3D surface of a simple and hard set of rules using Trap. MF.

**Table 4:** (A) The proposed simple set of fuzzy IF–then rules that take the decisions, (B) The proposed hard set of fuzzy IF–then rules that take the decisions.

*IF—and*		Economic loss					
		Notable	Minor	Serious	Major	Catastrophic	Disastrous
Safety							
(A)							
Notable	*Then*	Notable	Notable	Minor	Minor	Serious	Major
Minor		Notable	Minor	Minor	Serious	Serious	Major
Serious		Minor	Minor	Serious	Serious	Major	Catastrophic
Major		Minor	Serious	Serious	Major	Major	Catastrophic
Catastrophic		Serious	Serious	Major	Major	Catastrophic	Disastrous
Disastrous		Serious	Major	Catastrophic	Catastrophic	Disastrous	Disastrous
(B)							
Notable	*Then*	Notable	Minor	Serious	Major	Catastrophic	Disastrous
Minor		Minor	Minor	Serious	Major	Catastrophic	Disastrous
Serious		Serious	Serious	Serious	Major	Catastrophic	Disastrous
Major		Major	Major	Major	Major	Catastrophic	Disastrous
Catastrophic		Catastrophic	Catastrophic	Catastrophic	Catastrophic	Catastrophic	Disastrous
Disastrous		Disastrous	Disastrous	Disastrous	Disastrous	Disastrous	Disastrous

### Consent to participate

All authors consent to participating in this work.

## Results

The annual frequency of the initiating cause and PFD for each IPL (collected from the look-up tables, the offshore reliability data (OREDA), and several companies’ standards according to the industry experience)^11,22^. MEF is calculated by multiplying the frequency of initiating cause and the PFD of each IPL the result is compared with TEF to know if the suggested IPLs are enough for the potential hazard or if they need more risk reduction. NRR is calculated by dividing TEF/MEF the result presents the PFD of the SIS wanted to reduce the risk. Then we can calculate RRF is equal to PFD^−1^. Thus SIL rating can be calculated according to Table1. Table 5 shows the LOPA results sheet.

**Table 5:** LOPA results sheet and SIL rating.

S.N	Impact even and TEF (/year)	Cause and frequency (/year)	IPL (integrated protection layers) and its PFD (process failure on demand)				Extra mitigation measures include a fire safety system, pressure relief, restricted access, etc	Mitigated frequency of event (/year)	Reduced risk as needed (TEF/MEF)	Risk reduction factor (RRF = PFD-1)	SIL rating
			Process design	BPCS	Alarms, Procedures	SIS (PLC, relays)					
1	Potential for damage within the vulnerable (low pressure) discharge side of the existing crude oil pumps due to overpressure	High pressure/low pressure interface (HP/LP) between the new crude oil shipping pumps’ discharge side AND the existing crude oil pumps discharge side			High pressure alarms can predict the operator						
	1E−4	1E−1	–	–	1E−1	–	–	1E−2	1E−2	100	2
2	Potential for damage within the vulnerable (low pressure) discharge side of the existing crude oil pumps due to overpressure	Reverse flow from the new crude oil pumps’ discharge into the existing crude oil pumps’ discharge	Check valve on existing crude oil pumps discharge side		High pressure alarms can predict the operator						
	1E−5	65E−3	1E−1	–	1E−1	–	–	65E−5	1.5E−3	65	1

Applying fuzzy logic to the system using different membership functions to select the best one ,Results from the three models showed in Table 6A, B were compared and showed that trap. MF. gives the least RRF for the selected scenarios which can cause several damage in location and an economic loss.

**Table 6:** (A) Comparison between 3 different MF based on simple set of rules. (B) Comparison between 3 different MF based on hard set of rules.

E. lose	Safety hazard	GAUS.MF				TRAP.MF				TRIA.MF			
		Severity	TEF	GEF	RRF	Severity	TEF	GEF	RRF	Severity	TEF	GEF	RRF
(A)													
30 M$	1	− 3.64	2.3E−4	1E−2	43	− 3.54	2.88E−4	1E−2	35	− 3.6	2.5E−4	1E−2	40
500 M $	3	− 4.81	1.55E−5	65E−5	42	− 4.54	2.88E−5	65E−5	22	− 4.81	1.55E−5	65E−5	42
(B)													
30 M$	1	− 3.94	1.15E−4	1E−3	87	− 3.94	1.48E−4	1E−2	87	− 3.98	1.05E−4	1E−2	95
500 M $	3	− 4.95	1.12E−5	65E−5	58	− 4.81	1.55E−5	65E−5	42	− 4.96	1.09E−5	65E−5	60

Finally LOPA and fuzzy LOPA results are compared as illustrated in Table 7, which showed that in spite of being SIL is the same in traditional and fuzzy LOPA, fuzzy approach gives better results in risk reduction.

**Table 7:** Comparison between traditional and fuzzy logic LOPA.

Scenarios	Layers of protection analysis (LOPA)				Fuzzy logic							
					Simple rules				Hard rules			
	TEF	MEF	RRF	SIL	TEF	MEF	RRF	SIL	TEF	MEF	RRF	SIL
1. High pressure/low pressure interface (HP/LP) between the new crude oil shipping pumps’ discharge side AND the existing crude oil pumps discharge side	1E−4	1E−2	100	2	2.88E−4	1E−2	35	1	1.48E−4	1E−2	78	1
2. Reverse flow from the new crude oil pumps’ discharge into the existing crude oil pumps’ discharge	1E−5	65E−5	65	1	2.95E−5	65E−5	22	1	1.55E−5	65E−5	42	1

## Discussion

The previous results demonstrated the effectiveness of the proposed study in reducing the risk, whether in hard or simple rules using a one stage model instead of using a cascade fuzzy model as Khalil^14^. Using different models demonstrated the effectiveness of using the trapezoidal membership function over others to reduce the risk instead of guessing a special one as at^16^.

The proposed method is considered more effective in analyzing risks that cause serious damage than the qualitative method, which does not give us adequate analysis of such risks and in a way simpler than the quantitative method, integration of the proposed method with the existing safety management systems in petroleum production facilities will lead to effective results in saving time and money. Using fuzzy logic model may have a higher cost than using the LOPA model, but considering the impact of its use of reducing the RRF this model is considering more economical, this can be explained by looking at the results in the scenario of high pressure/low pressure interface (HP/LP) between the new crude oil shipping pumps’ discharge side and the existing crude oil pumps discharge side, as in the safety integrity level change from SIL1 at LOPA model to SIL2 at the fuzzy model, the cost of applying safety measures at SIL2 much higher than in SIL1.which is much lower than the cost of applying fuzzy logic on the system. This illustrates the economic impact of the proposed study. The proposed method is considered more effective in analyzing risks that cause serious damage than the qualitative method, which does not give us adequate analysis of such risks and in a way that is less complex than the quantitative method. Integration of the proposed method with the existing safety management systems in petroleum production facilities will lead to effective results in saving time and money. The results of the mentioned scenarios shows the sensitivity of the study as each one has different inputs which causing change in the RRF value obtained.

## Conclusions

The process of crude oil transportation is considered the first step in the petroleum industry. Therefore, applying means of protection and risk analysis is extremely important to limit the occurrence of any problems that may hinder the transportation process and may cause accidents. In this study, the major risk caused by reverse flow from the new crude oil pumps’ discharge into the existing crude oil pumps’ discharge was studied and layers of protection analysis (LOPA) were used as a semi-quantitative method for risk analysis after determining it using the qualitative method (HAZOP). The proposed method determines the SIL rating in order to identify the process need for any additional means of protection.

Fuzzy logic technology has been used to determine SIL and validate the obtained results from the LOPA study in a faster and more economical way that serves well in the planning process for establishing any industrial facility. This fuzzy model has been established based on economic loss and number of injuries that can be produced from the potential hazard using the most common membership functions (Gaussian, trapezoidal, and triangular) and using two rule assumptions (simple &hard). Since fuzzy systems’ performance is totally based on experts and their contributions towards setting up the membership functions, the rules, and the defuzzification method, several trials have been considered. The obtained results for two fuzzy models are shown. According to obtained results, it could be noted that both the LOPA and the fuzzy LOPA give convergent results in calculating the SIL rating. Especially if the RRF value is in the middle of the level range, but if it is on the level’s border, applying fuzzy logic takes us to the lower level in either the simple or hard rules. Furthermore, the use of trapezoidal membership functions has given the most adequate results.

Applying fuzzy logic technique can produce good results if used in quantitative risk analysis if the safety integrity level of the system results to be SIL3 or SIL4 with simple and cost effective method.

## Data Availability

All data generated or analysed during this study are included in this published article. Data supporting reported results can be found in the papers included in the References section.
